# Effect of Gemcitabine based chemotherapy on the immunogenicity of pancreatic tumour cells and T-cells

**DOI:** 10.1007/s12094-020-02429-0

**Published:** 2020-07-13

**Authors:** P. L. Smith, Y. Yogaratnam, M. Samad, S. Kasow, A. G. Dalgleish

**Affiliations:** ST Georges University of London, 1 Cranmer Terrace, London, SW17 0RE UK

**Keywords:** Pancreatic cancer, Chemotherapy, Immunotherapy, Gemcitabine

## Abstract

**Purpose:**

Chemotherapy for advanced pancreatic cancer has limited efficacy due to the difficultly of treating established tumours and the evolution of tumour resistance. Chemotherapies for pancreatic cancer are typically studied for their cytotoxic properties rather than for their ability to increase the immunogenicity of pancreatic tumour cells. In this study Gemcitabine in combination with immune modulatory chemotherapies Oxaliplatin, zoledronic acid and pomalidomide was studied to determine how combination therapy alters the immunogenicity of pancreatic tumour cell lines and subsequent T-cell responses.

**Methods:**

Pancreatic tumour cell lines were stimulated with the chemotherapeutic agents and markers of immune recognition were assessed. The effect of chemotherapeutic agents on DC function was measured using uptake of CFSE-stained PANC-1 cells, changes in markers of maturation and their ability to activate CD8+ T-cells. The effect of chemotherapeutic agents on T-cell priming prior to activation using anti-CD3 and anti-CD28 antibodies was determined by measuring IFN-γ expression and Annexin V staining using flow cytometry.

**Results:**

These agents demonstrate both additive and inhibitory properties on a range of markers of immunogenicity. Gemcitabine was notable for its ability to induce the upregulation of human leukocyte antigen and checkpoints on pancreatic tumour cell lines whilst inhibiting T-cell activation. Pomalidomide demonstrated immune modulatory properties on dendritic cells and T-cells, even in the presence of gemcitabine.

**Discussion:**

These data highlight the complex interactions of different agents in the modulation of tumour immunogenicity and immune cell activation and emphasise the complexity in rationally designing chemo immunogenic combinations for use with immunotherapy.

## Introduction

Despite recent advances in cancer treatment, systemic chemotherapy still remains the only option for unresectable or metastatic pancreatic cancer. Folinic acid, fluorouracil, irinotecan and Oxaliplatin [1] (FOLFORINOX) or Gemcitabine (GEM) in combination with Paclitaxel [2] are the main chemotherapeutic options available however due to the toxicities associated with these drugs GEM alone has typically been prescribed in approximately 46% of patients [3]. Even with greater first line options approximately 32% of patients receive gemcitabine monotherapy as first or second line treatment [4]. Pancreatic cancer quickly develops resistance to these chemotherapeutic regimens, limiting their efficacy. The median survival after pancreatic cancer diagnosis is approximately 4.6 months [5] which emphasises the urgent need for more effective treatment options. Thus, new chemotherapeutic regimens are required with improved efficacy [6].

The clinical potential of immunotherapy is being realised through the use of checkpoint inhibitors (CPI) and engineered T-cells [7]. However, with very few exceptions, these immunotherapeutic approaches have not been particularly successful when applied to pancreatic cancer [8], likely due to the late presentation of the disease by which point the tumour is often locally advanced with a well-developed immune suppressive microenvironment [9]. CPI in combination with single agent GEM or GEM plus Paclitaxcel has repeatedly failed to show significant efficacy in pancreatic cancer patients [10–16] indicating that new chemotherapeutic approaches are needed for subsequent combination with immunotherapy.

Chemotherapeutic combinations for pancreatic cancer have typically been studied for their cytotoxic properties rather than their ability to increase the immunogenicity of pancreatic tumour cells. Our group has previously demonstrated immune modulatory properties of GEM [17] indicating that it may be a suitable chemotherapeutic with which to develop novel combinations of immune modulating chemotherapeutic agents in the setting of pancreatic cancer. In this study chemotherapeutic agents Pomalidomide (POM), Oxaliplatin (OXP) and Zoledronic acid (ZA) were selected to be paired with GEM on the basis of their varied immune modulatory properties [18–21]. In this study the effect of these GEM-based combinations was assessed in vitro on markers of immunogenicity in pancreatic tumour cell lines, dendritic cells and T-cells.

## Methods

### Reagents

Gemcitabine, Oxaliplatin, Pomalidomide and ZAedronic acid, Staphylococcus enterotoxin B (SEB) and WST-1 reagent were purchased from Sigma (Dorset, UK). Flow cytometry antibodies were purchased from Biolegend (London, UK) and R and D systems (Abingdon, UK). Cytomegalovirus, Epstein Barr Virus and Influenza virus (CEF) peptides were purchased from Sigma (Dorset, UK). CFSE reagent was purchased from Fisher Scientific—UK Ltd (Loughborough, UK).

### Cell culture

Pancreatic tumour cell lines PANC-1, Miapaca-2 and BxCP-3 were cultured in Dulbecco’s modified eagles medium (DMEM) with 10% FBS and 5% Penicillin/Streptomycin at 37 °C, 5% CO_2_. Leukocyte cones obtained from the NHS national blood service (London, UK). PBMC were isolated using Histopac purchased from Sigma (Dorset, UK) as described previously [22].

### Cytotoxicity assay

WST-1 viability assays were performed as detailed in the manufactures protocol. Briefly, pancreatic tumour cell lines were seeded into flat bottom 96 well plates at a concentration of 5 × 103 cells in 100 μl of DMEM. After overnight adherence the tumour cells were treated with the indicated concentration of chemotherapeutic agents and incubated for a further 72 h. Supernatant was removed prior to incubation of the cells in WST-1 reagent (Sigma, Dorset, UK) for 20 min. Colour change was assessed using a spectrometer at 490 nm.

### Generation of MDDC and T-cells

MDDC were generated by isolating CD14+ cells using MACS CD14 isolation beads (Miltenyi, Bergisch Gladbach, Germany). The isolated cells were incubated with IL-4 and GMCSF for 6 days as previously described [22]. T-cells were isolated by positive selection using MACS CD3 isolation beads according to the manufacturer’s instructions.

### Flow cytometry

Pancreatic tumour cell lines, MDDC and T-cells were assessed for expression of different markers using the following antibodies: Anti-PDL-1 APC, anti-Galectin 9 PE, anti-CD39 FITC, anti-CD47 Percp-Cy5.5, anti-HLA-class I Alexa Flour 700, anti-MIC A/B PE, anti-ULBP1 FITC, anti-ULBP 2,5 6 Percp, anti-ULBP 3 APC,anti-CD86 PE, anti-CCR7 PE-CY7, anti-CD40 APC, anti-HLA-class II PE-CY5 anti-IFN-γ PE-CY7, anti-CD3 Alexa Flour 488, anti-CD4 APC-CY7, anti-CD8-PE and anti-CD69 APC (Biolegend, CA).

### Tumour cell uptake assay

PANC-1 tumour cells were incubated with 1 µM of CFSE reagent overnight. Cells were subsequently washed in PBS and incubated with indicated concentration of chemotherapeutic agents for 24 h. The cells were washed twice and incubated with 1 × 10^5^ MDDC for 4 h. The non-adherent MDDC were stained with an PE-CY5 anti-human HLA-class II antibody and assessed for uptake of CFSE labelled PANC-1 cells by flow cytometry (Biolegend, CA).

### MDDC maturation

MDDC were incubated with supernatant from treated or untreated pancreatic tumour cell lines for 24 h prior to staining with antibodies specific for CD83, CD86, CCR7, PDL-1, HLA-class I, CD40 and HLA-class II (Biolegend, CA)..

### T-cell activation

DC were matured as described above and loaded with CEF peptide or SEB antigen prior to co-culture with autologous CD14 negative PBMC. Brefeldin A was added after 18 h and the cells were incubated for a further 6 h prior to staining with antibodies for anti-human-CD3 FITC, anti-human, anti-human CD8-PE, anti-human IFN-γ-PE-CY7. Purified T-cells were also directly treated with combinations of chemotherapeutic agents for 24 h and stained for anti-human CD69–APC. To measure the onset of activation induced cell death (AICD) anti-PD-1 (1–10 μg/ml), anti-CD3 (5 μg/ml) and anti-CD28 (2.5 μg/ml) antibodies were used to stimulate T-cells prior to staining with anti-IFN-γ PE-CY7 and Annexin V APC (Biolegend, CA).

## Results

### Markers of immunogenicity in pancreatic cell lines

**Fig. 1:**
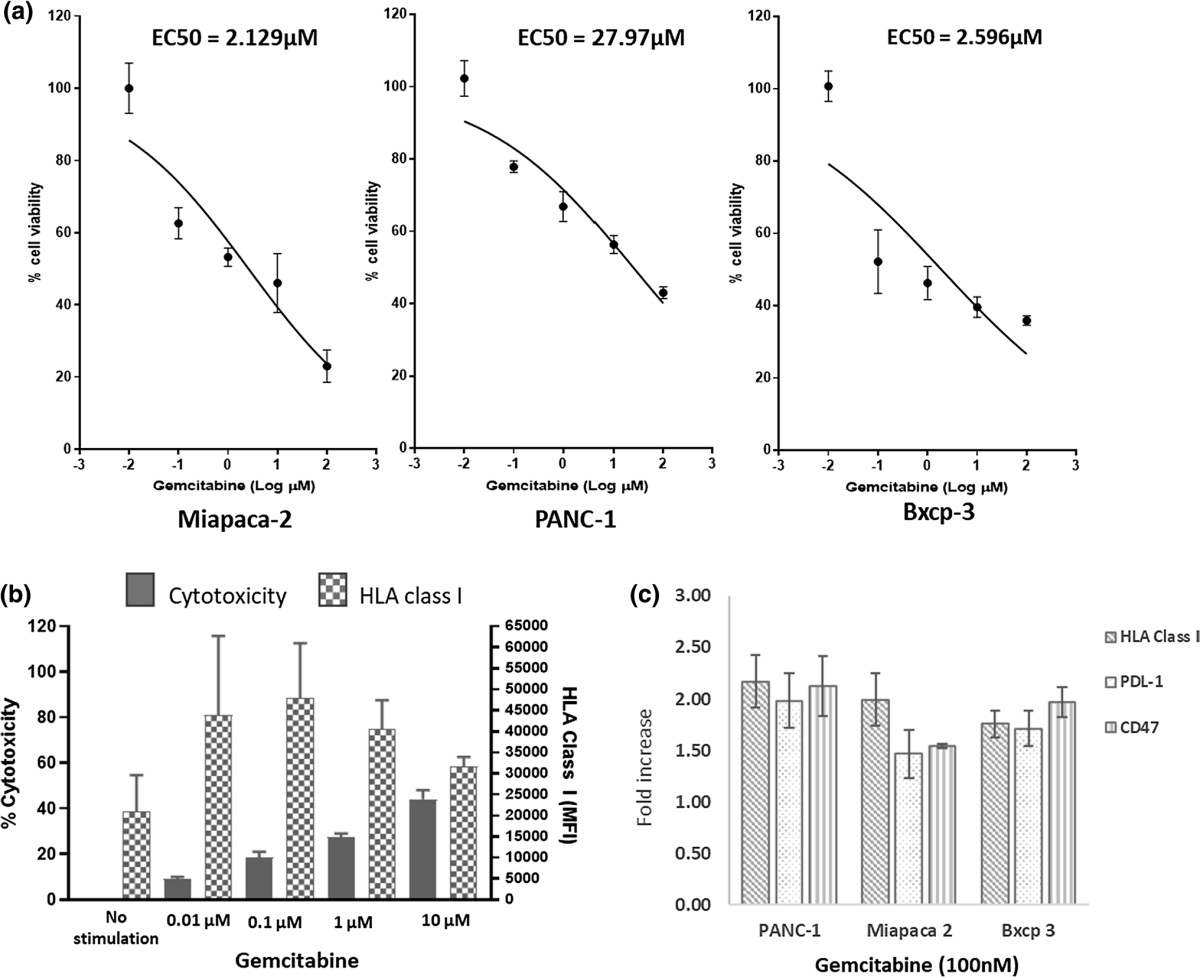
Effect of GEM on cytotoxicity and markers of tumour recognition. GEM (0.01–10 μM) was incubated with each pancreatic tumour cell line for 72 h prior to the addition of WST-1 reagent to measure cell viability (**a**). Expression of HLA (48 h) and cytotoxicity (72 h) of PANC-1 cells by different concentrations of GEM (**b**). Fold increase in the expression of HLA-class I, PDL-1 and CD47 by GEM in each cell line after 24 h incubation (**c**). *N* = a minimum of 3 experiments

Cytotoxicity by the selected chemotherapeutic agents against three pancreatic tumour cell lines PANC-1, Miapaca-2 and Bxcp-3, was assessed (Fig. 1; Table 1). Consistent with previous studies GEM was cytotoxic towards these tumour cell lines which in turn demonstrated different susceptibility to GEM-based killing (Fig. 1a; Table 1). Combinations of GEM with either ZA or OXP had an additive effect on tumour cell cytotoxicity, lowering GEM’s EC50 (Table 1). In contrast POM only demonstrated a discernible cytotoxic effect either alone or in combination with GEM against Miapaca-1 cells (Table 1) consistent with our previous work combining GEM with lenalidomide, an immune modulatory drug similar to POM [23].

**Table 1:** EC50 of different chemotherapeutic agents on PANC-1, Miapaca-2 and BxCP-3 cell lines

Agent	EC50 µM (95% CI) of agents against pancreatic tumour cell lines		
	PANC-1	Miapaca-2	Bxcp-3
Gemcitabine (GEM)	24.97 (9–38.4)	2.5 (1–7.1)	1.90 (0.4–11.8)
Oxaliplatin (OXP)	4.52 (3.2–6.5)	5.01 (1.5–22.3)	4.52 (3.2–6.5)
Zoledronic acid (ZA)	1.04 (0.5–21.4)	0.14 (0.06 to 0.2)	1.34 (0.6–2.76)
Pomalidomide (POM)	NR	4.01 (1–22.6)	NR
Gem + OXP (1 µM)	0.81 (0.05–9.9)	1.43 (0.7–3.9)	0.73 (0.1–6.8)
Gem + ZA (100 nM)	1.96 (0.88–4.8)	7.48 (1.8–52.2)	0.81 (0.05–9.9)
GEM + POM (10 µM)	20.65 (5.17–31.8)	2.84 (0.8–13)	1.31 (0.1–21)

**Fig. 2:**
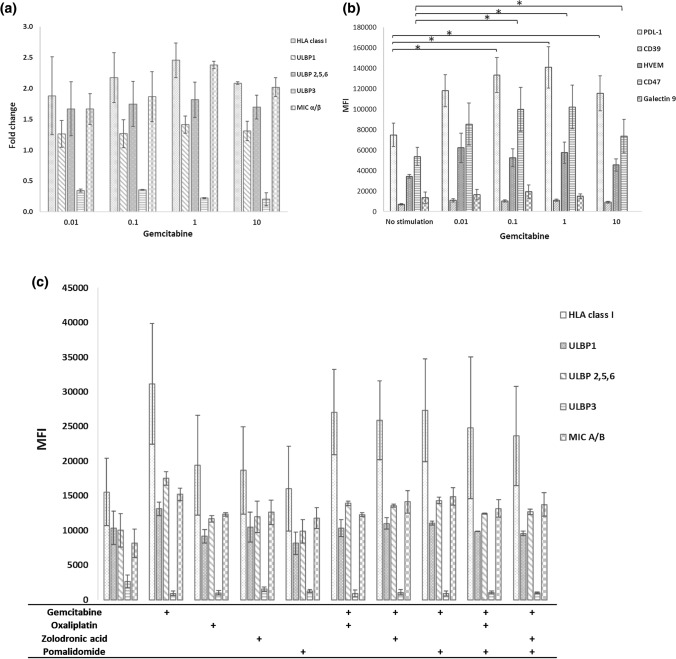
GEM-based combination treatment and markers of tumour recognition. PANC-1 cells were treated for 48 h with different concentrations of GEM and changes in the expression of markers of tumour recognition HLA-class I, ULBP1, 3, 2/5/6 and MIC A/B (a) or immune checkpoints CD39, HVEM, PDL-1 (0.1 µM *p* = 0.007, 1 µM *p* = 0.006, 1 µM *p* = 0.014) Galectin 9 and CD47 (0.1 µM *p* = 0.007, 1 µM *p* = 0.006, 1 µM *p* = 0.05) were measured (**b**). *N* = 3. GEM (100 nM), OXP (100 nM), ZA (10 nM) and POM (100 nM) were used alone or in combination to stimulate PANC-1 cells for 48 h and markers of tumour recognition were measured by flow cytometry. *N* = 3. Paired *t* tests were performed to assess statistical significance

The ability of GEM to increase HLA-class I expression on pancreatic tumour cell lines was assessed. GEM increased HLA expression after 48 h stimulation of each of the cell lines studied (Fig. 1b, c). Interestingly increases in HLA appeared to be independent of the ability of GEM to kill tumour cells (Fig. 1b) occurring at sub-optimal concentrations (10–100 nM) to induce cytotoxicity and at a time point before which cytotoxicity is observed. Incubation of GEM with each cell line increased the expression of HLA-class I, PDL-1 and CD47 on each tumour cell line (Fig. 1c). Concentrations of GEM (10 nM) unable to induce cytotoxicity were able to increase the expression of ULBP1, 2, 5 and 6 in addition to MIC A/B (Fig. 2a). In contrast GEM stimulation resulted in a decrease in ULBP3. The ability of GEM to alter the expression of immune checkpoints was also assessed, of five checkpoints measured GEM significantly increased the expression of PDL-1 and CD47 in each cell line studied but had no effect on the expression of CD39, Galectin 9 or HVEM (Figs. 1c, 2b). Neither OXP, ZA or POM could increase the expression of HLA-class I, ULBP’s, MIC A/B but didn’t significantly alter the ability of GEM to upregulate these receptors (Fig. 2). These data indicate that, of the agents studied, the ability to increase the expression of markers of tumour recognition was restricted to GEM.

**Fig. 3:**
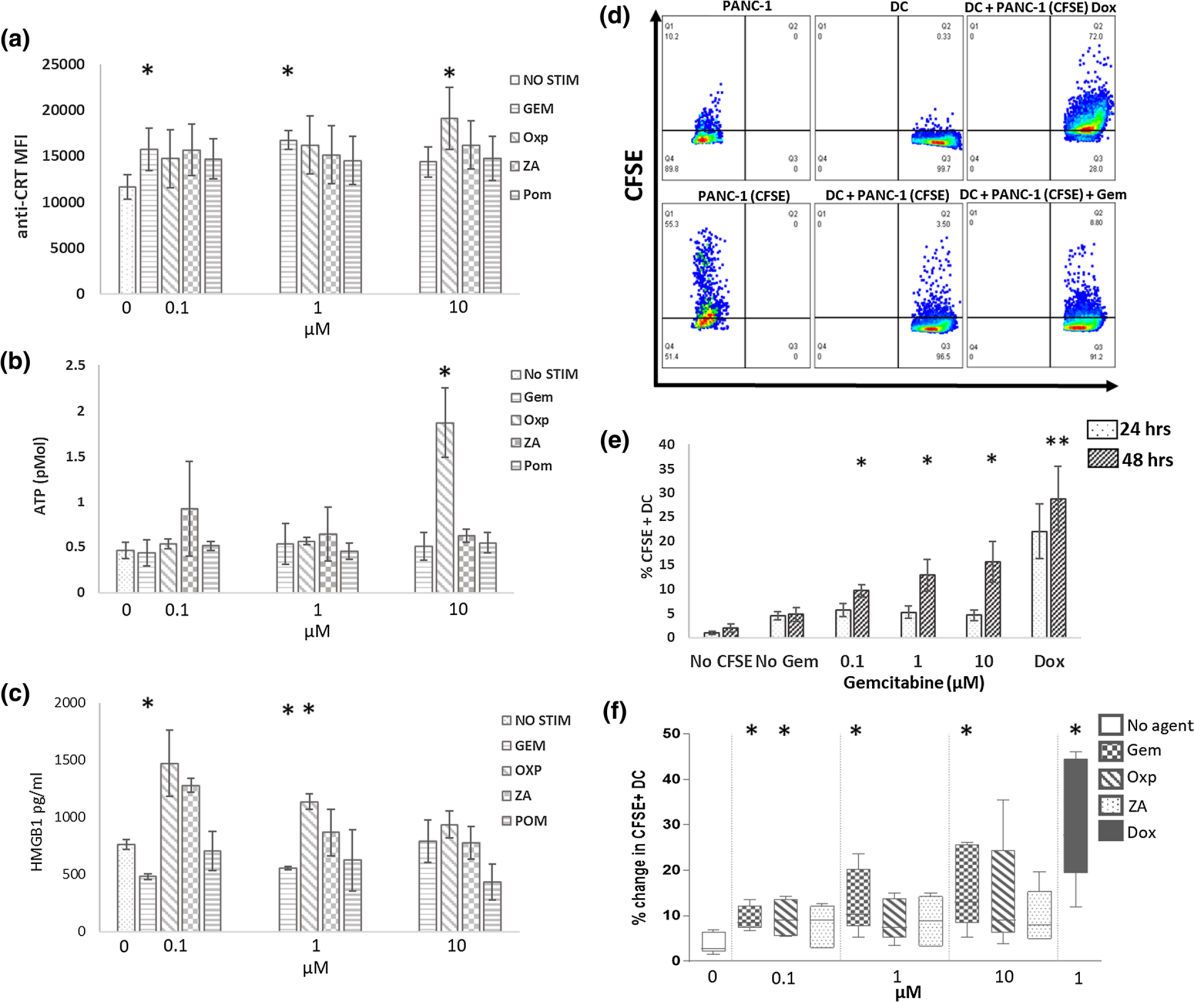
Induction of markers of immunogenic cell death from chemotherapeutic agents. Calreticulin (**a**) (0.1 µM GEM *p* = 0.04, 1 µM GEM *p* = 0.04; 1 µM OXP *p* = 0.016), ATP (**b**) (10 µM OXP *p* = 0.05) and HMGB1 (**c**) (0.1 µM GEM *p* = 0.0073, 1 µM GEM *p* = 0.013; 1 µM OXP *p* = 0.018) were measured in PANC-1 cells stimulated with different chemotherapeutic agents for 24–48 h. *N* = at least 3 experiments. A paired *t* test was used to determine statistical significance over unstimulated control. CFSE-stained PANC-1 cells treated with chemotherapeutic agents for 24 or 48 h were incubated for 4 h with MDDC and uptake was measured by flow cytometry. Incubation of CFSE + PANC-1 cells with chemotherapeutic agents promotes their internalisation by HLA-DR hi MDDC (**d**). Effect of uptake of PANC-1 cells at 24 and 48 h stimulation GEM (0.1 µM GEM *p* = 0.008, 1 µM GEM *p* = 0.027; 10 µM GEM *p* = 0.029) (**e**). GEM and OXP but not ZA promote PANC-1 uptake by DC (**f**)

Next, the ability of each chemotherapeutic agent to induce markers of ICD from pancreatic tumour cell lines was determined. GEM demonstrated an ability to induce the cell surface expression of Calreticulin on PANC-1 tumour cell lines (Fig. 3a). Similar effects were observed with Miapaca-2 and Bxcp-3 cells (data not shown). OXP, an established inducer of ICD was also capable of promoting CRT translocation whereas neither ZA or POM induced observable CRT translocation. Secretion of ATP and HMGB1 are also established markers of ICD [24]. GEM, ZA and POM were unable to induce the expression ATP or HMGB1, in contrast to OXP (Fig. 3b, c). Combinations of chemotherapeutic agents had no additive effect on markers of ICD (data not shown).

### Effect of chemotherapeutic agents on monocyte derived dendritic cells

PANC-1 cells were used to study the effect of GEM on functional responses from immune effector cells (Fig. 3e). First, CFSE-stained PANC-1 cells were stimulated with either single agent or GEM-based combinations for 24 and 48 h prior to co-culture with monocyte derived dendritic cells (MDDC) for 4 h (Fig. 3d, e). Stimulation of PANC-1 cells tended to increase their uptake into MDDC with statistically significant increases observed for GEM (Fig. 3d) and OXP but not ZA (Fig. 3f). Combinations of agents did not increase uptake of PANC-1 cells into MDDC (data not shown).

**Fig. 4:**
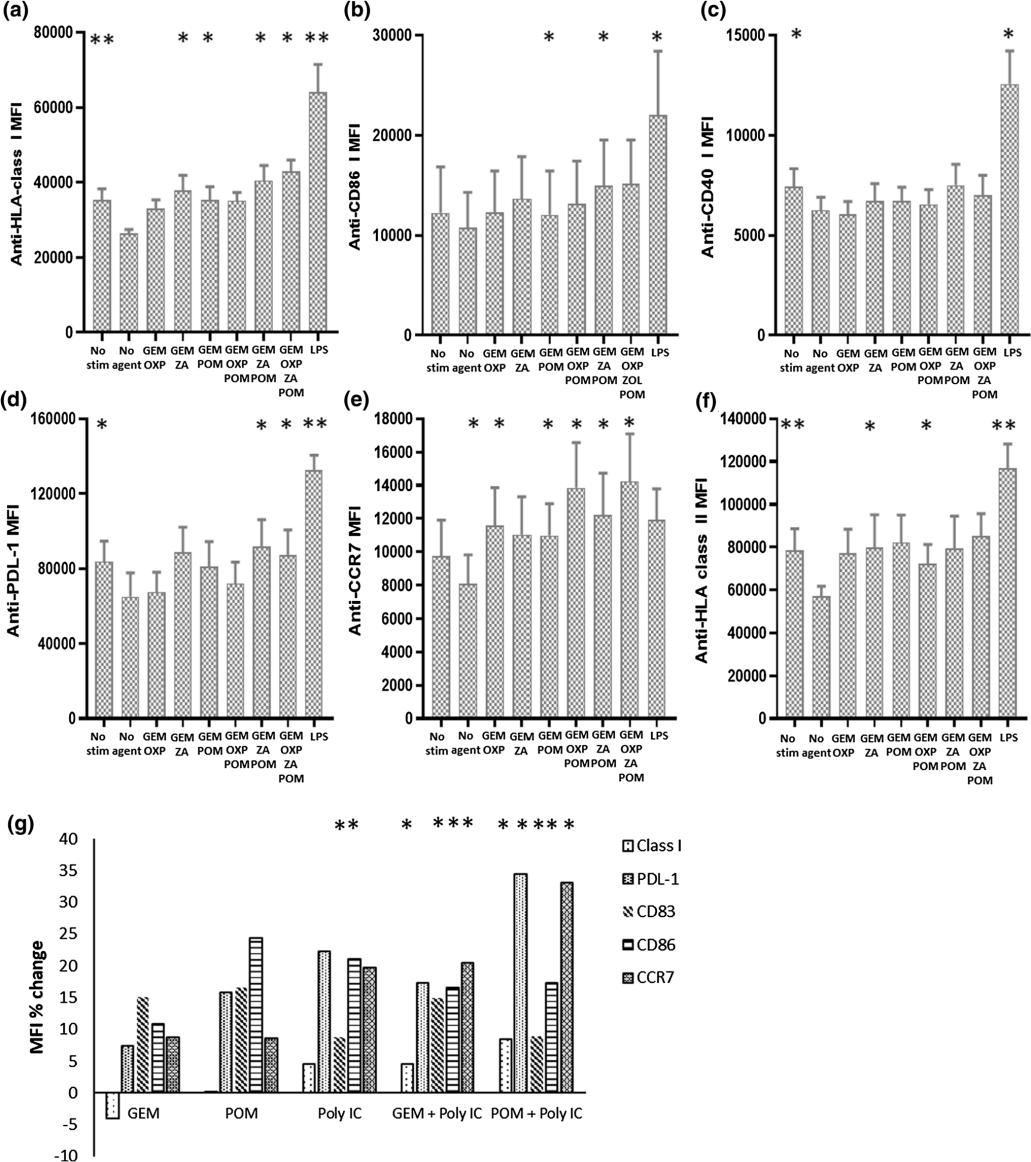
GEM-based combinations enhance DC maturation: Immature MDDC were cultured with supernatant from pancreatic tumour cells treated with GEM-based combinations or left untreated. No supernatant (No stimulation) and LPS were utilised as controls. Flow cytometry was used to assess markers of maturation after 24 h HLA-class I (**a**), CD86 (**b**), CD40 (**c**), PDL-1 (**d**), CCR7 (**e**) and HLA-class II (**f**). *N* = experiments from 6 different donor PBMC. GEM (10 nM) or POM (10 nM) were paired with Poly IC (1 μg/ml) and incubated with MDDC for 24 h and markers of maturation were assessed (**g**). Paired *t* tests were used to assess statistical significance

We sought to determine whether treatment of pancreatic tumour cells with chemotherapy alters the maturation of MDDC exposed to their supernatants and whether direct incubation of MDDC with chemotherapeutic agents effects their maturation. Incubation of MDDC with supernatant from PANC-1 cells significantly reduced the expression of HLA-class I, HLA-class II, PDL-1 and CD40 compared to untreated MDDC (Fig. 4a, c, d, f). CCR7 and CD86 expression was not significantly altered. The supernatant of PANC-1 cells treated with single agents had no effect on any marker with the exception of supernatant from POM treated PANC-1 cells which significantly increased the expression of CD40 compared to that of supernatant from untreated PANC-1 cells.

Treatment of PANC-1 cells with combinations of chemotherapeutic agents significantly increased the expression of MDDC markers of maturation. HLA-class I, class II, CD86 and CCR7, but not CD40, expression could be significantly increased by incubation of MDDC with supernatants from PANC-1 cells treated with GEM-based combinations containing ZA, POM and/or OXP (Fig. 4a, b, d–f) compared to supernatant from untreated PANC-1 cells. Next, MDDC were directly stimulated with chemotherapeutic agents and markers of maturation were assessed. Each agent, alone or in combination, significantly increased the expression of HLA-class I compared to the untreated control. In addition, combinations of GEM including ZA or POM significantly increased the expression of CD86 and CD40 respectively (data not shown). However, CCR7, HLA-class II, and PDL-1 were unchanged.

Stimulation of MDDC with 100 ng/ml of the TLR4 agonist LPS significantly increased the expression of each marker with the exception of CCR7 when compared to untreated or PANC-1 supernatant incubated MDDC. To assess the effect of TLR ligation in combination with chemotherapeutic agents Poly IC, a TLR3 agonist, was combined with single agent GEM, which had demonstrated no effect on DC maturation or POM which had demonstrated limited effects on DC maturation (Fig. 4e). Poly IC plus either GEM or POM resulted in increases in markers of MDDC maturation compared to the no stimulation control (Fig. 4g).

### Effect of chemotherapeutic agents on T-cell responses

**Fig. 5:**
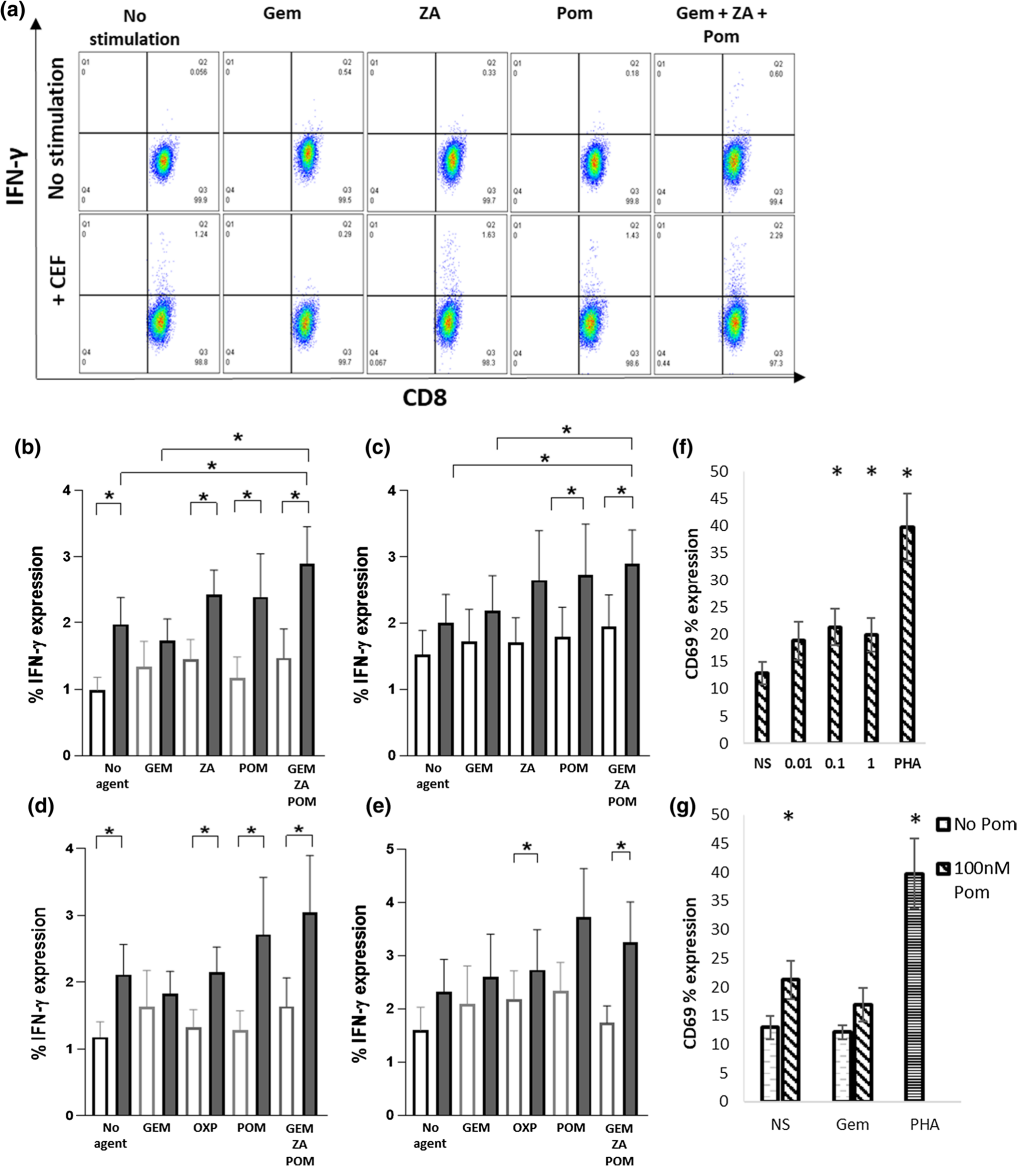
Treatment with combinations of chemotherapeutic agents significantly increases antigen specific IFN-γ expression from CD8+ T-cells. IFN-γ expression from CD8+ T-cells stimulated with treated MDDC expressing CEF epitopes (**a**). CD8+ T-cells were stimulated with MDDC incubated with supernatant from PANC-1 cells treated with GEM, ZA or POM (**b**) or with MDDC directly stimulated with GEM, ZA, POM (**c**) and loaded with CEF peptide. CD8+ T-cells were stimulated with MDDC incubated with supernatant from PANC-1 cells treated with GEM, OXP or POM (**d**) or with MDDC directly stimulated with GEM, OXP, POM (**e**) and loaded with CEF peptide. *N* = at least 6 experiments. Isolated T-cells were stimulated with POM for 24 h and CD69 expression on CD8+ T-cells was measured by flow cytometry (**f**). CD69 expression from CD8+ T-cells stimulated for 24 h with GEM ± POM (**g**). *N* = 4

**Fig. 6:**
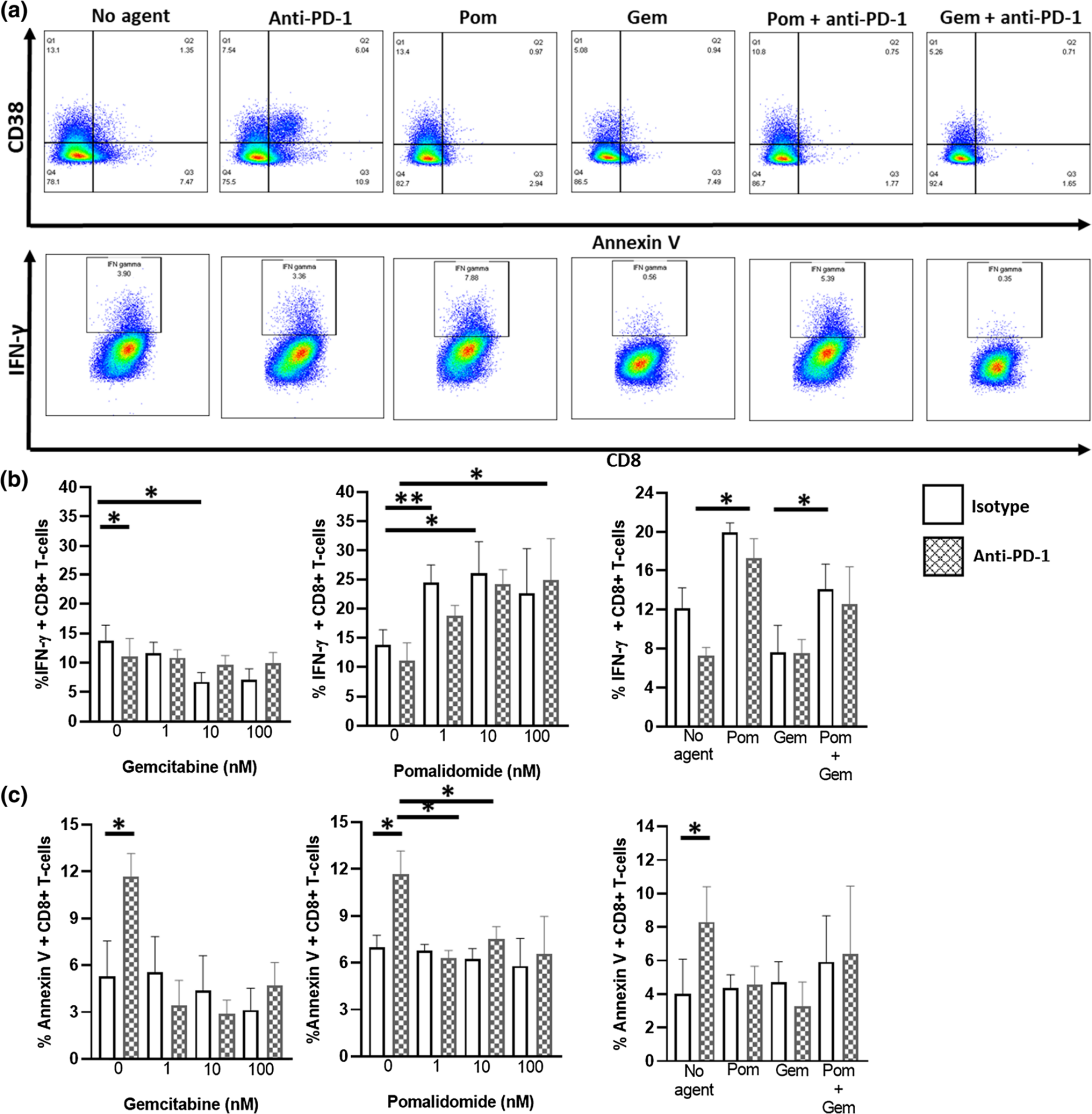
Pomalidomide modulates activation of CD8 + T-cells pre-incubated with anti-PD-1 antibody. T-cells were incubated with anti-PD-1 (10 μg/ml) ± GEM (1–100 nM) or POM (1–100 nM) or combinations of GEM (10 nM) and POM (10 nM) for 48 h prior to activation with anti-CD3 and anti-CD28 antibodies for a further 48 h (**a**). Effect of Anti-PD-1 pre-stimulation ± POM or GEM on IFN-γ expression (**b**), or Annexin V expression (**c**) from CD8+ T-cells. *N* = 4

Next, the ability of treated MDDC to stimulate antigen specific CD8+ T-cell responses was assessed (Fig. 5). MDDC incubated with supernatant from treated PANC-1 tumour cells or directly stimulated with chemotherapeutic agents for 24 h were co-cultured with a peptide pool containing immunodominant epitopes from cytomegalovirus, Epstein Barr virus and influenza virus (CEF) and co-cultured with PBMC for a further 24 h. Intracellular cytokine staining was used to assess the antigen specific expression of IFN-γ from CD8+ T-cells (Fig. 5). Consistent with the ability of combination agents to induce MDDC maturation, combinations including GEM and POM with either ZA or OXP significantly increased the expression of IFN-γ from CD8+ T-cells. Single agent POM was able to stimulate increased CEF dependent expression of IFN-γ from CD8+ T-cells whereas single agent GEM reduced IFN-γ expression (Fig. 5b, c). To assess the direct effect of POM on T-cell activation, isolated T-cells were incubated with POM (1–100 nM) and CD69 expression was assessed (Fig. 5g). POM significantly increased the expression of CD69 on CD8+ T-cells (Fig. 6f). Combining POM with GEM showed that whilst GEM had no significant effect on CD69 expression it could partially block the effect of POM. Taken together these data suggest an inhibitory effect of GEM on T-cell activation.

CD8+, CD101+ T-cells expressing CD38 and PD-1 have been associated with poor prognosis in pancreatic cancer [25]. CD101 is a marker of T-cell exhaustion [26] and CD38 and PD-1 co-expressing T-cells are thought to be dysfunctional [27]. We sought to determine whether GEM pre-stimulation followed by T-cell activation could cause increases in CD38 and PD-1 expression on T-cells. Activation of T-cells with anti-CD3 plus anti-CD28 antibodies increased the expression of CD38 and PD-1; however, preincubation with gemcitabine resulted in no additional expression either CD4+ or CD8+ T-cell subsets. POM significantly increased the expression of IFN-γ from effector and central memory CD8+ T-cell populations, consistent with its ability to increase IFN-γ expression from CEF stimulated T-cells (Fig. 6) but did not significantly increase the expression of CD38 and PD-1 (data not shown). The frequency of CD101 on T-cell subsets was not changed by preincubation with POM or GEM and the expression of CD38 and PD-1 on CD101+ cells was also unchanged.

Finally, checkpoint blockade with anti-PD-1 antibodies has been implicated in the onset of dysfunction and apoptosis of T-cells associated with poor prognosis in PDAC [27]. Consistent with these findings we found that preincubation of anti-PD-1 antibody (10 μg/ml) followed by T-cell activation resulted in increases in Annexin V staining and reductions in IFN-γ expression in CD8+ T-cells (Fig. 6). The ability of GEM or POM to co-stimulate T-cells in the presence of anti-PD-1 antibody was measured. Incubation of T-cells with GEM + anti-PD-1 antibody prior to T-cell activation resulted in reductions in both IFN-γ and Annexin V staining (Fig. 6b, e). In contrast incubation of T-cells with POM + anti-PD-1 antibody reduced the expression of Annexin V staining compared to anti-PD-1 incubation alone whilst increasing the expression of IFN-γ in CD8+ T-cells (Fig. 6c, f) but not CD4+ T-cells. Incubation of Pom with GEM + anti-PD-1 antibody was able to recapitulate IFN-γ expression in CD8+ T-cells but was unable to reduce the expression of Annexin V (Fig. 6d, g).

## Discussion

Improving the immune modulatory properties of GEM-based therapy will benefit pancreatic cancer patients by providing more effective chemo-immunotherapy based treatments capable of killing tumour cells through direct cytotoxic effects and by supporting anti-tumour immune responses activated with immunotherapies such as checkpoint inhibition [28, 29].

This study demonstrates the complexity of combining different agents with varied effects on diverse markers of the immune response. Of the agents studied here GEM was unique in its ability to upregulate markers of tumour recognition from pancreatic tumour cell lines including HLA-class I, MIC A/B and ULBP receptors (Figs. 1, 2). It would be interesting to ascertain whether GEM can enhance the targeting of pancreatic tumour cells by effector cells capable of recognising MIC A/B and ULBP such as NK and γδ-T-cells. GEM was the only agent studied which upregulated checkpoint molecules including PDL-1 and CD47. This has relevance for its potential as an immunotherapeutic agent in combination with anti-PD-1 or anti-PDL-1 inhibitors whilst implicating these checkpoints in blocking putative immune potentiating properties of GEM in vivo. Notably GEM could induce increases in HLA expression at concentrations inducing minimal cytotoxicity (Fig. 1) indicating that the immunogenic and cytotoxic properties of GEM may be independent. These data have implications for the use and dose of GEM in different therapeutic settings.

In contrast GEM was unable to induce expression of all three markers of ICD. Oxaliplatin, capable of ICD, increased the expression of CRT, ATP and HMGB1 from pancreatic tumour cell lines, in line with previous studies [30]. GEM increased the expression of CRT on the surface of pancreatic tumour cells and enhanced the uptake of PANC-1 cells into DC but could not induce the expression of ATP or HMGB1 from the cell lines studied (Fig. 3). Given the ability of GEM to increase the expression of the checkpoint CD47 which is involved in blocking the CRT dependent uptake of tumour cells it would be interesting to ascertain whether blocking CD47 expression on PANC-1 cells further enhanced their GEM mediated uptake into APC’s.

Combination treatment of PANC-1 cells induced factors that could significantly increase the expression of markers of DC maturation compared to no treatment controls or single agent GEM (Fig. 4). These increases were lower compared to LPS or Poly IC but were associated with a significantly increased ability of these DC to activate antigen specific IFN-γ expression from CD8+ T-cells (Fig. 5). The increases in both DC maturation and T-cell activation were likely due in part to the presence of the iMiD POM in the combination treatment, consistent with our previous findings [20]. GEM alone was unable to either induce DC maturation or activation of T-cell responses. GEM, but not OXP or ZA, was associated with inhibition of T-cell activation (Figs. 5, 6) suggesting a potential role for GEM in T-cell dysfunction.

Dysfunctional T-cell subsets, defined by expression of PD-1 and CD38 are associated with poor prognosis in GEM treated pancreatic patients, particularly on CD101+ expressing T-cells which represent an exhausted phenotype that cannot be salvaged by anti-PD-1 therapy [26]. A recent study indicated that CD38^hi^, PD-1^hi^ T-cells are susceptible to apoptosis upon interaction with anti-PD-1 antibody prior to T-cell priming [27]. Incubation with GEM and/or POM did not significantly alter the expression of these markers upon stimulation of T-cell subsets from healthy donors. However, GEM inhibited IFN-γ expression from T-cells which could be partially restored with POM co stimulation (Fig. 6d). POM was also capable of reducing the onset of markers of apoptosis and increase the expression of IFN-γ from T-cells incubated with anti-PD-1 antibody prior to T-cell activation (Fig. 6b, e) consistent with the ability of POM to prime T-cell responses.

Its notable that GEM had the greatest effect on markers of tumour recognition, OXP on markers of ICD and POM on the priming of T-cell immune responses. Combinations of these agents rarely demonstrated additive or inhibitory properties, with the exception of GEM dependent inhibition of T-cell responses. Although several chemotherapeutics have well defined immunogenic effects their clinical efficacy has rarely been associated with the onset of immune responses, even for known inducers of ICD. A possible explanation for the limited observable immunotherapeutic effects of chemotherapeutic agents is that the promotion anti-tumour immune responses involves a multitude of checkpoints and effector cells whilst a single agent such as GEM mediates only a subset of these factors. The agents studied here demonstrated modest ability to induce markers of ICD (Fig. 3) or DC maturation (Fig. 4), suggesting that this component of chemo-immunotherapy needs to be addressed. Combinations involving chemotherapy and broad immune stimulants including TLR agonists such as Poly IC [31, 32] or cytokines such as IFN-α [33] have shown promise and the combination of GEM or POM with Poly IC studied here demonstrated increased DC maturation (Fig. 4g). The potential therapeutic benefit of combination of chemotherapy with TLR agonists is illustrated by our recent study demonstrating improved responses in PDAC patients with the addition of heat killed supported *Mycobacterium obuense* to single agent GEM [34].

A greater understanding of the immunological effects of combination chemotherapy, in addition to factors such as dose and sequence, is likely needed to improve immunotherapy in cancers such as pancreatic cancer. These data highlight that chemotherapeutics such as GEM can benefit by the addition immune modulators capable of inducing strong DC maturation and T-cell activation. It will be interesting to study the effect of these combinations in vivo during which the activation of tumour recognition and ICD, DC maturation and activation of T-cell responses may demonstrate cumulative anti-tumour effects which are not possible to study using the in vitro assays described here.

Checkpoint inhibition has demonstrated poor efficacy against pancreatic cancer. Priming with immune modulatory agents [34] followed by immunogenic chemotherapy such as with GEM plus POM may promote greater effectiveness of checkpoint inhibition. We have previously reported on a complete response (>2 years) in a case study of metastatic pancreatic cancer involving treatment with enalidomide, GEM and a heat killed preparation of *Mycobacterium vaccae*. In a more recent case study a complete response was observed involving GEM and *Mycobacterium obuense* by the CPI Pembrolizumab (unpublished observation). In conclusion, this study has demonstrated distinct in vitro immune modulatory effects of GEM and POM on pancreatic tumour cell lines and T-cells respectively. This indicates that these agents are suitable for combination with immunotherapy such as checkpoint inhibition, particularly alongside innate immune agonists capable of promoting immunogenic cell death or DC maturation.
